# Cost-Effectiveness of Blood Donation Screening for *Trypanosoma cruzi* in Mexico

**DOI:** 10.1371/journal.pntd.0004528

**Published:** 2016-03-22

**Authors:** Gilberto Sánchez-González, Alejandro Figueroa-Lara, Miguel Elizondo-Cano, Leslie Wilson, Barbara Novelo-Garza, Leopoldo Valiente-Banuet, Janine M. Ramsey

**Affiliations:** 1 Immunology Division, National Institute of Public Health, Cuernavaca, Mexico; 2 Escuela Militar de Graduados de Sanidad, Mexico City, Mexico; 3 Division of Innovation and Technology Management, Mexican Social Security Institute, Mexico City, Mexico; 4 Health Economics Division, National Institute of Public Health, Cuernavaca, Mexico; 5 Departments of Medicine and Pharmacy, University of California, San Francisco, San Francisco, California, United States of America; 6 Medical Infrastructure Planning Coordination, Mexican Social Security Institute, Mexico City, Mexico; 7 Centro de Ciencias de la Complejidad C3, UNAM, Mexico City, Mexico; 8 Regional Center for Public Health Research, National Institute for Public Health Research, Tapachula, Chiapas, Mexico; Oswaldo Cruz Foundation, BRAZIL

## Abstract

An estimated 2 million inhabitants are infected with Chagas disease in Mexico, with highest prevalence coinciding with highest demographic density in the southern half of the country. After vector-borne transmission, *Trypanosoma cruzi* is principally transmitted to humans via blood transfusion. Despite initiation of serological screening of blood donations or donors for *T*. *cruzi* since 1990 in most Latin American countries, Mexico only finally included mandatory serological screening nationwide in official Norms in 2012. Most recent regulatory changes and segmented blood services in Mexico may affect compliance of mandatory screening guidelines. The objective of this study was to calculate the incremental cost-effectiveness ratio for total compliance of current guidelines from both Mexican primary healthcare and regular salaried worker health service institutions: the Secretary of Health and the Mexican Institute for Social Security. We developed a bi-modular model to analyze compliance using a decision tree for the most common screening algorithms for each health institution, and a Markov transition model for the natural history of illness and care. The incremental cost effectiveness ratio based on life-years gained is US$ 383 for the Secretary of Health, while the cost for an additional life-year gained is US$ 463 for the Social Security Institute. The results of the present study suggest that due to incomplete compliance of Mexico’s national legislation during 2013 and 2014, the MoH has failed to confirm 15,162 *T*. *cruzi* infections, has not prevented 2,347 avoidable infections, and has lost 333,483 life-years. Although there is a vast difference in *T*. *cruzi* prevalence between Bolivia and Mexico, Bolivia established mandatory blood screening for *T*.*cruzi* in 1996 and until 2002 detected and discarded 11,489 *T*. *cruzi* -infected blood units and prevented 2,879 potential infections with their transfusion blood screening program. In the first two years of Mexico’s mandated program, the two primary institutions failed to prevent due to incomplete compliance more potential infections than those gained from the first five years of Bolivia’s program. Full regulatory compliance should be clearly understood as mandatory for the sake of blood security, and its monitoring and analysis in Mexico should be part of the health authority’s responsibility.

## Author Summary

Chagas disease continues to be a neglected disease in Mexico and Latin-American. Although an estimated 96% of *Trypanosoma cruzi* transmission to humans occurs via 32 triatomine vector species, the only transmission prevention in Mexico has been sparse and based on heterogeneous blood donation screening. Despite mandating serological screening of blood donations or donors for *T*. *cruzi* since 1990 in most Latin American countries, Mexico only finally included mandatory serological screening nationwide in official Norms in 2012. In 2005, a survey of blood donor centers in Mexico was conducted to compare *T*. *cruzi* prevalence in donations with that of Mexican migrants in the US. Since there was little coincidence between data from that survey and official screening or confirmed case rates, and screening for the social security system only initiated in 2010, the objective of this study was to calculate the incremental cost-effectiveness ratio for total compliance of current guidelines from both Mexican primary healthcare (the Secretary of Health) and regular salaried worker health services (the Mexican Institute for Social Security). A bi-modular model to analyze compliance was developed using a decision tree for the most common documented screening algorithms for the two principal health institution, and a Markov transition model for the natural history of illness and care. The incremental cost effectiveness ratio based on life-years gained is US$ 383 for the Secretary of Health (MoH), while the cost for an additional life-year gained is US$ 463 for the Social Security Institute (IMSS). Using survey compliance data for MoH, and that published by IMSS, failure to detect current infections, to avoid new infections, and life-years lost were calculated for 2013 and 2014 for both institutions. The MoH has failed to confirm 15,162 *T*. *cruzi* infections, did not prevent 2,347 avoidable infections, and lost 333,483 life-years over the two year period. Full regulatory compliance should be mandatory and timely monitoring should be part of the health authority’s responsibilities for the sake of blood security in Mexico.

## Introduction

Chagas disease is caused by the unicellular parasite *Trypanosoma cruzi*, capable of movement directly from one person to another via blood transfusion, organ transplant, or maternal-fetal transfer [1, 2]. Although the most prevalent mode of transmission is via the excreta of infected reduviid bugs, where vectors are not present, iatrogenic trypanosomiasis is considered the most important [3–5]. An estimated minimum 10 million individuals are infected worldwide with corresponding incidence of 41,200 cases per year [6]. Approximately 99% of inhabitants infected with Chagas disease (CD) reside in Latin America, where between 25 and 90 million persons are at infection risk via one of the multiple infection modes. The disease burden for CD in the Latin American and Caribbean region, based on disability-adjusted life-years (DALYs) is five times greater than malaria, and is approximately one-fifth that of HIV/AIDS [6, 7].

Despite overall prevalence estimates for the Latin American region, there are an estimated 1.1 to 2 million Mexicans infected with *T*. *cruzi* [8–11], with highest estimated prevalence in the southern half of the country [12]. Rural to urban population migrations in the last decades, have provoked largely unplanned urban development and landscape modifications surrounding cities, which are important amplifiers of zoonotic hosts and pathogens, and improved opportunities for 32 triatomine species to persist [12]. More than half of the *T*. *cruzi* infected vector-exposed Mexican population now lives in urban areas. Infected inhabitants are rarely diagnosed for *T*. *cruzi* infection since there is an overall lack of epidemiological surveillance for its transmission or for disease, and if an infection is detected due to blood donation screening, patients are rarely treated with anti-parasitic drugs [13]. Clinical and public health personnel have little knowledge regarding Chagas disease (CD), its transmission, clinical diagnosis, or treatment, due to neglect by healthcare system policies. Most individuals with *T*. *cruzi* infection or Chagas disease (CD) are asymptomatic or symptomatic without clinical recognition of etiology (cardiac insufficiency or megaviscera), and unaware, as are healthcare personnel, of potential blood transfusion risk [14]. Third level hospitals in Mexico City report from 0.37% (National Institute of Cardiology) [15, 16] to 0.17% (National Institute of Pediatrics) [17] of blood donations with antibody to *T*. *cruzi*. In contrast, 7.7% of blood donations from the Puebla Mexican Institute for Social Security (IMSS) have antibodies to *T*. *cruzi* [18]. In some Mexican blood banks, *T*. *cruzi* seroprevalence is higher than that of HIV, Hepatitis B, and Hepatitis C, corresponding more closely to the high seroprevalence detected in Mexican populations in the US [19–21]. There are twice as many blood donations from urban (> 10,000 inhabitants) as compared to rural populations in Mexico, which implies the need to adjust overall seroprevalence accordingly when these estimates are extrapolated to open population. The vast majority (> 90%) of *T*. *cruzi* infections in Mexico are in fact detected by blood donation screening, with the exception of those cases detected by research groups.

Interrupting blood transfusion of *T*. *cruzi* depends upon effective donor or blood donation screening. Guidelines formulated in 1994 by Mexico’s national legislation, the “Official Mexican Standard for disposition with therapeutic aims of human blood and its components (NOM-003-SSA2-1993)”, mandated blood screening for *T*. *cruzi* “if” donors resided in CD endemic areas [22]. However, endemic areas were not defined by this legislation, and at that time little if any cases were reported due to a lack of epidemiological surveillance. Most recent guidelines (NOM-253-SSA2-2012) replace those from 1994, and now mandate nationwide *T*. *cruzi* blood donation screening, using tests with at least 95% sensitivity and specificity, as established by the National Institute for Diagnostics and Epidemiological Reference (Instituto Nacional de Diagnóstico y Referencia epidemiologica, InDRE) [23]. Positive blood units detected by screening tests are discarded for therapeutic use, although they must be tested with two tests by approved reference laboratories. There has been no evaluation of the impact of the new guidelines on screening efficacy, costs, life-years gained, or CD case detection (epidemiological or clinical follow-up). The objective of the present study has been to fill that gap and analyze the impact of complete vs. incomplete compliance of the new guidelines for the Secretary of Health (MoH) and for the Mexican Institute for Social Security (IMSS). Combined, these two institutions attend approximately 70% of the Mexican population [24], while the former is also normative and heads the primary prevention and health care programs for vector-borne diseases in the country.

## Methods

**Fig 1:**
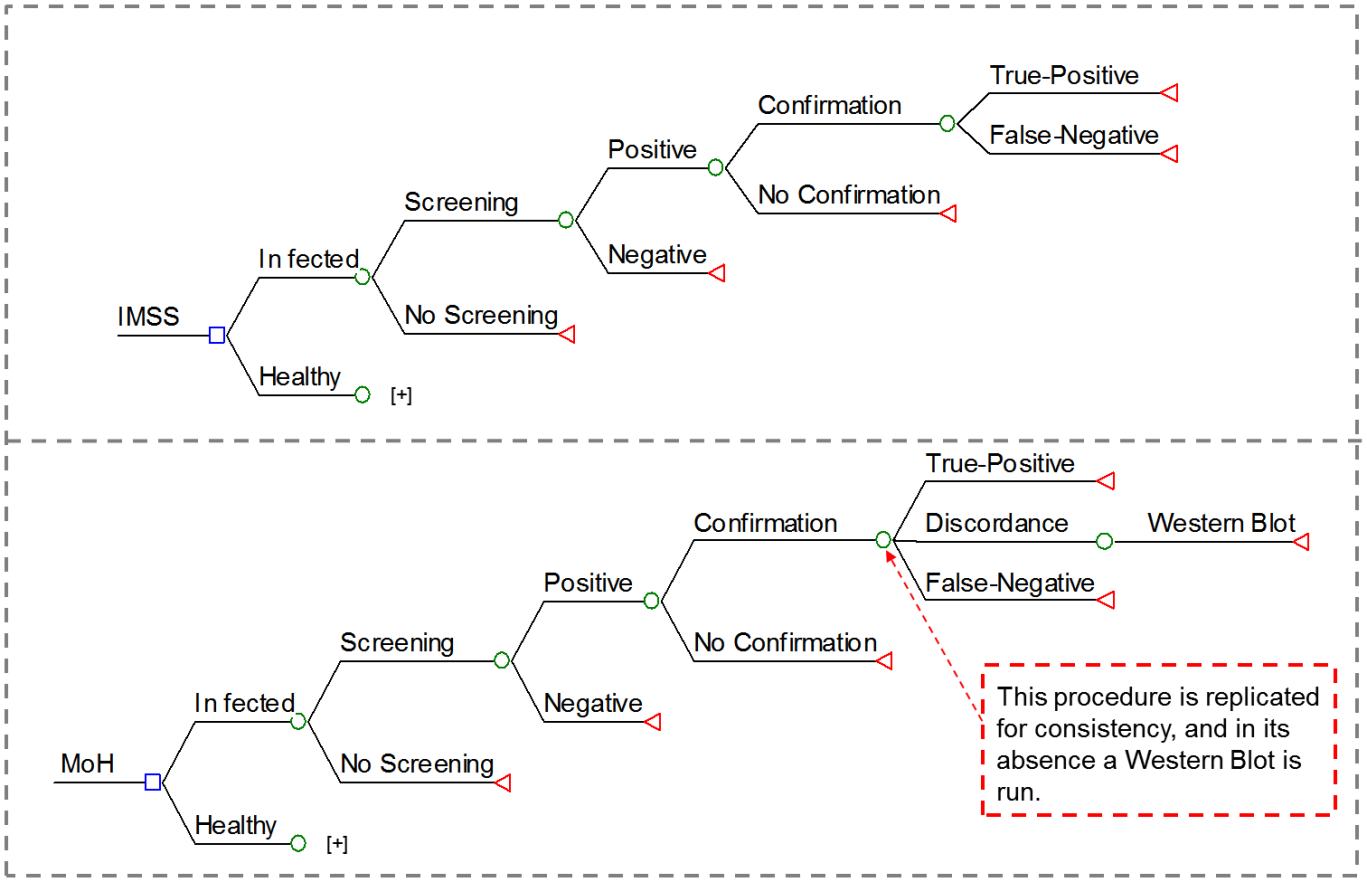
Decision trees used to simulate blood screening for MoH and IMSS scenarios.

Two scenarios were developed for each health institution, based on current documented estimates, and for 100% compliance (NOM-253-SSA2-2012). The first scenario reflects the known status of non-compliance, assigned based on donation center response to a screening questionnaire conducted in 2007 and categorized as “not all are screened and not all positives are confirmed”. The second scenario considers complete compliance of current guidelines from the category “all are screened and all positives are confirmed”. An analytical model for compliance and costs was constructed using two modules: 1) a decision tree for the most common donation screening algorithm based on most common practices at donation centers from each institution, and 2) a Markov transition model simulating the natural history of the illness and a standard care protocol for both institutions. The model of natural and/or clinical evolution of the illness is an application of the model developed previously by the group [11]. Professional software was used to construct the models (TreeAge Software, Williamstown, Massachusetts), the first of which is divided into two parts: decision trees from each blood donation center where a screening assay is conducted, and the follow-up procedure for confirmation of positive samples. The model structure of decision trees for both health institutions are illustrated in Fig 1.

Confirmation of MoH positive donations is conducted at state public health diagnostic laboratories, and all positive samples, in addition to 10% of negatives, are retested at the InDRE for quality control (National Institute for Diagnostics and Reference, a part of MoH, located in Mexico City). Confirmatory tests for IMSS samples are run in-house at one of four centralized reference laboratories (two in Mexico City, one in Guadalajara, and one in Monterrey). A decision tree was developed separately for each institution, since confirmation procedures were not the same. Parameters for each scenario and health institution are summarized in Table 1.

**Table 1:** Scenarios and decision tree model parameters, for each health institution.

Institution	Scenarios	Variable	Value	Source of data
Ministry of Health	First scenario	Proportion of blood screened	40%	[25]
		Proportion of *T*. *cruzi* positive blood sent for confirmation	39%	[26]
	Second scenario	Proportion of blood screened	100%	[23]
		Proportion of *T*. *cruzi* positive blood sent for confirmation	100%	[23]
		Indirect immunofluorescence (Architect Abbott)		
	Screening tests	Specificity	99.90%	[27]
		Sensitivity	96.60%	[27]
		Cost*	57	[28]
		Crude antigen ELISA (Chagatest Wiener Lab)		
		Specificity	98.90%	[29]
		Sensitivity	98.90%	[29]
		Cost	3.19	[30]
		Recombinant antigen ELISA (ChagasScreen Plus)		
		Specificity	98.70%	[31]
		Sensitivity	99.30%	[31]
		Cost	6.5	[32]
	Confirmatory tests	Indirect Hemagglutination Test (Interbiol)		
		Specificity	99.90%	[33]
		Sensitivity	99.90%	[33]
		Cost	39.4	[34]
		Crude antigen ELISA (Chagatest Wiener Lab)		
		Specificity	98.90%	[29]
		Sensitivity	98.90%	[29]
		Cost	3.19	[30]
		Western Blot (bioMérieux)		
		Specificity	97.30%	[35]
		Sensitivity	100%	[35]
		Cost	174	[28]
Mexican Social Security Institute	First scenario	Proportion of blood screened	87%	[36]
		Proportion of *T*. *cruzi* positive blood sent for confirmation	99%	[37]
	Second scenario	Proportion of blood screened	100%	[23]
		Proportion of *T*. *cruzi* positive blood sent for confirmation	100%	[23]
	Screening tests	Chemiluminescence (PRISM Abbott)		
		Specificity	99.80%	[38]
		Sensitivity	99.90%	[38]
		Cost	3.1	[37]
		Recombinant antigen ELISA (ChagasScreen Plus)		
		Specificity	98.70%	[31]
		Sensitivity	99.30%	[31]
		Cost	6.5	[32]
	Confirmatory test	Lysate ELISA (BioChile Chagas ELISA II),		
		Specificity	95.30%	[39]
		Sensitivity	99.30%	[39]
		Cost*	3.1	[37]
Parameters of the population and infectivity				
Variable			Value	Source of data
Chagas prevalence			0.0123	[26]
Average age of donors			33	[18]
Average age of recipients			45	[40]
Probability of infection due to an infected blood unit			0.18	[41]

**Fig 2:**
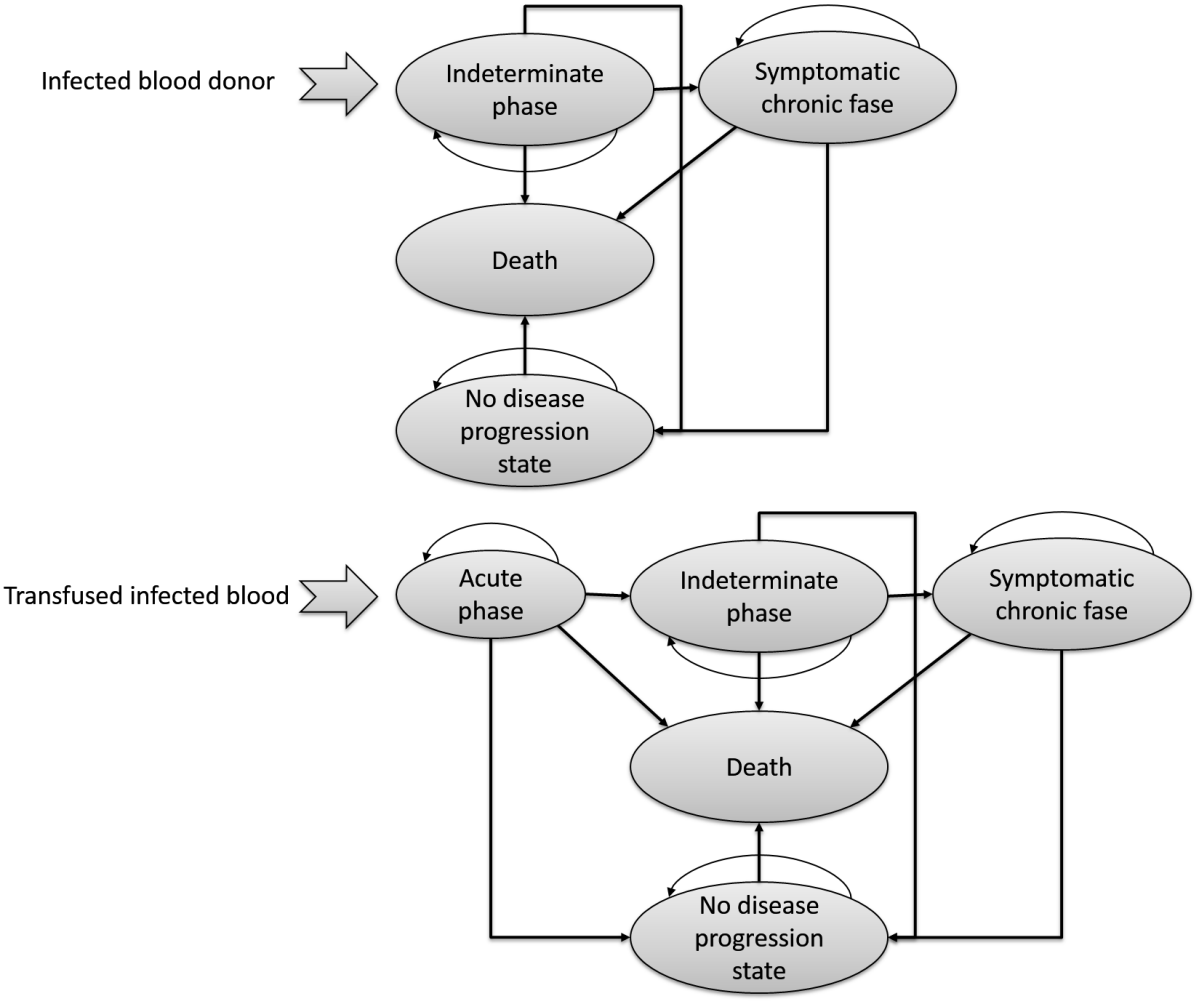
General structure of the Markov model with all clinically important events and transition pathways, from one state to another.

Infected recipients of undetected blood units enter the natural history of the disease, modeled by the Markov transition module. The model assumes that infected donors are in the indeterminate asymptomatic phase of CD (or their health status would have excluded them upon initial screening interview) and that the prevalence of infected donors is the same as that of the general population. Independent of whether an infected individual has or not been diagnosed for *T*. *cruzi*, the person enters an additional Markov model module for disease evolution [11]. This Markov module has five health phases: acute, chronic asymptomatic, symptomatic chronic phase, no progression phase, and death. Each time-step length is one month in the acute phase and one year for later phases. Changes in time steps are managed as follows: in the acute stage, each time step represents one month by introducing monthly transition probabilities, whereas the time of life accumulated, runs as 1/12 per cycle. Similarly, the discount rate runs with the time divided by 12. The chronic asymptomatic, symptomatic chronic, and no progression phases are driven by annual transition probabilities and with annual accumulated time of life, as well as the discount rates. A middle-step correction was introduced in the model. Infected donors in the asymptomatic phase, are randomly distributed across the average duration of the phase. Infected recipients enter the model in the acute phase. The simulation runs until the entire cohort dies (Fig 2). Donors in the model are characterized by age and their infection status (apriori assigned); a person may be either uninfected (truly not infected with *T*. *cruzi*) or infected (truly infected with *T*. *cruzi*). The infection status of donors is assigned randomly based on national population prevalence. The model identifies blood units as true positives or as false negatives, depending on results from the screening tests. Donors to be screened are selected randomly based on the screening rates for each scenario. If a donor is detected positive, the model assumes that the person will begin specific anti-parasitic drug treatment. When a person is infected by an undetected infected blood unit, both the donor and the recipient remain undiagnosed and continue with the natural history of the disease.

Screening tests modeled for MoH were those most frequently used by donor centers and reported to the National Blood Transfusion Center. These were a recombinant antigen indirect immunofluorescence assay (Architect Abbott), a recombinant antigen ELISA (ChagasScreen Plus), and a crude antigen ELISA (Chagatest Wiener Lab) [42]. Screening tests modeled for the IMSS were chemiluminescence (PRISM Abbott) and a recombinant antigen ELISA (ChagasScreen Plus), which were the most frequently used by in-house donation centers and registered with the IMSS Medical Infrastructure Planning Coordination [43]. Donors with positive results in the screening tests were randomly selected for confirmatory tests based on the confirmation rate for each scenario. The confirmation procedure for MoH (InDRE) consisted of two simultaneous tests, a crude antigen ELISA and an indirect hemagglutination test (Interbiol). If the tests were discordant, both were run a second time, and if the tests persisted discordant, a Western Blot test (bioMérieux) was run. The criterium for a positive sample was that two out of three tests be positive. The confirmation test for IMSS was a single lysate ELISA (BioChile Chagas ELISA II).

The comparative performance between current and complete compliance was analyzed by comparing Chagas-specific mortality, new infections produced, and the incremental cost-effectiveness ratio of life-years gained. Percentage of blood screening by the Mexican Institute of Social Security was 87% and its confirmation rate was 99%, whereas for the Ministry of Health 40% of donations screened and 39% confirmed (based on a 2007 survey). Total cost is the sum of direct costs for medical care and indirect costs. Only the monetary value of work days lost was considered based on a modified social perspective. All costs are expressed as the 2014 value of the US dollar. Effectiveness variables generated were life-years gained and cases detected. Both costs and effectiveness variables were discounted at 5% per year.

A second order Monte Carlo simulation was used to simulate a cohort of 100,000 donors with 500 different sets of parameters for recalculations; 100,000 donor screening outcomes were obtained from 500 replicates using random sampling of the distributions assigned to each parameter. All parameters used to feed the model were introduced as statistical distributions: cost inputs are set as gamma distributions and the effectiveness and probabilities of transition are beta distributed. The Monte Carlo method, an alternative to analyze sensitivity, first selects a random set of input data values drawn from their individual probability distributions. These values are then used in the simulation model to obtain certain model output variable values. The result is a probability distribution of model output variables and system performance indices which result from variations and possible values of all input values [44–45]. Since all distributions are sampled in a Second Order Monte Carlo calculation, no independent sensitivity analysis was necessary.

## Results

The sum of costs for screening and confirmation tests, healthcare, and labor costs due to work days lost for detected and undetected cases, and blood costs per 100,000 donors, is US$ 23.2 million dollars for the MoH. Healthcare and labor costs of undetected cases are 62.9% of the total cost, 18.3% correspond to healthcare and labor costs of detected cases, 18% to blood cost, and the remaining to screening and confirmation tests. If there is complete compliance, the total cost is US$ 31.6 million, 36% greater than incomplete compliance. Healthcare and labor costs of detected cases represent 83.8% of the total cost for 100% compliance (Table 2). The total cost of the current compliance for IMSS is US$ 32.7 million, 71.6% of which is due to healthcare and labor costs of detected cases, 12.8% due to blood donation costs, 7.9% to healthcare and labor costs of undetected cases, and 7.7% due to screening and confirmation tests. The cost of complete compliance for IMSS is US$ 34.3 million, 5% greater than current incomplete compliance (Table 2).

**Table 2:** Average total cost and confidence interval (95%) for 100,000 blood donations per cost category, health institution, and coverage scenario.

Cost category (2014 US$)					
	Cost for screening and confirmatory tests	Healthcare and labor cost of detected cases	Healthcare and labor cost of undetected cases	Blood cost	Total costs
Secretary of Health					
First scenario (status quo)	183,749 (154,389–225,596)	4,263,546 (4,050,369–4,476,723)	14,606,282 (7,303,141–21,909,423)	4,185,537 (2,092,768–6,278,305)	23,239,114 (13,600,667–32,890,048)
Second scenario (100%)	580,996 (502,457–679,544)	26,502,135 (23,851,921–29,152,348)	360,982 (180,491–541,473)	4,185,537 (2,092,768–6,278,305)	31,629,649 (26,627,637–36,651,669)
Mexican Social Security Institute					
First scenario (status quo)	2,528,453 (2,507,545–2,548,013)	23,494,602 (22,319,872–24,669,332)	2,586,275 (1,293,138–3,879,413)	4,185,537 (2,092,768–6,278,305)	32,794,867 (28,213,323–37,375,062)
Second scenario (100%)	2,906,269 (2,900,173–2,912,068)	26,978,175 (24,280,357–29,675,992)	313,719 (156,860–470,579)	4,185,537 (2,092,768–6,278,305)	34,383,700 (29,430,159–39,336,944)

Effectiveness for all compliance scenarios and for both institutions are summarized in Table 3. In the current scenario for MoH, 190 cases are confirmed, there are 157 new *T*. *cruzi* infections detected, and 4,195 life-years are gained. If the MoH attains 100% compliance, 1,185 cases are confirmed (1,105% increase), 3 new *T*. *cruzi* infections are identified (154 new *T*.*cruzi* infections avoided), and 26,079 life-years are gained, which is 5.2 times greater the life-years gained. A 15% increase in the number of confirmed cases identifies 28 additional *T*. *cruzi* infections avoided (93.3%), and 15% of life-years gained were identified from complete compliance in IMSS.

**Table 3:** Average effectiveness and confidence interval (95%) per 100,000 blood donors and incremental cost-effectiveness ratio (ICER) according to health institution and coverage scenario. All costs in 2014 US$.

	Effectiveness category			ICER	
	Number of confirmed case (range)	New *T*. *cruzi* infections (range)	Life years gained due to diagnosis (range)	Cost per detected case	Cost per year of life gained
MoH					
First scenario (status quo)	190 (181–201)	157 (141–174)	4,195 (3,992–4,412)	54,483 (52,312–57,084)	383(325–401)
Second scenario (100%)	1,185 (1,067–1,304)	3 (0–5)	26,079 (23,463–28,677)		
IMSS					
First scenario (status quo)	1,050 (998–1,103)	30 (29–32)	23,119 (21,945–24,255)	56,744 (53,962–59,426)	463(420–493)
Second scenario (100%)	1,206 (1,085–1,327)	2 (0–5)	26,547 (23,879–29,185)		

**Fig 3:**
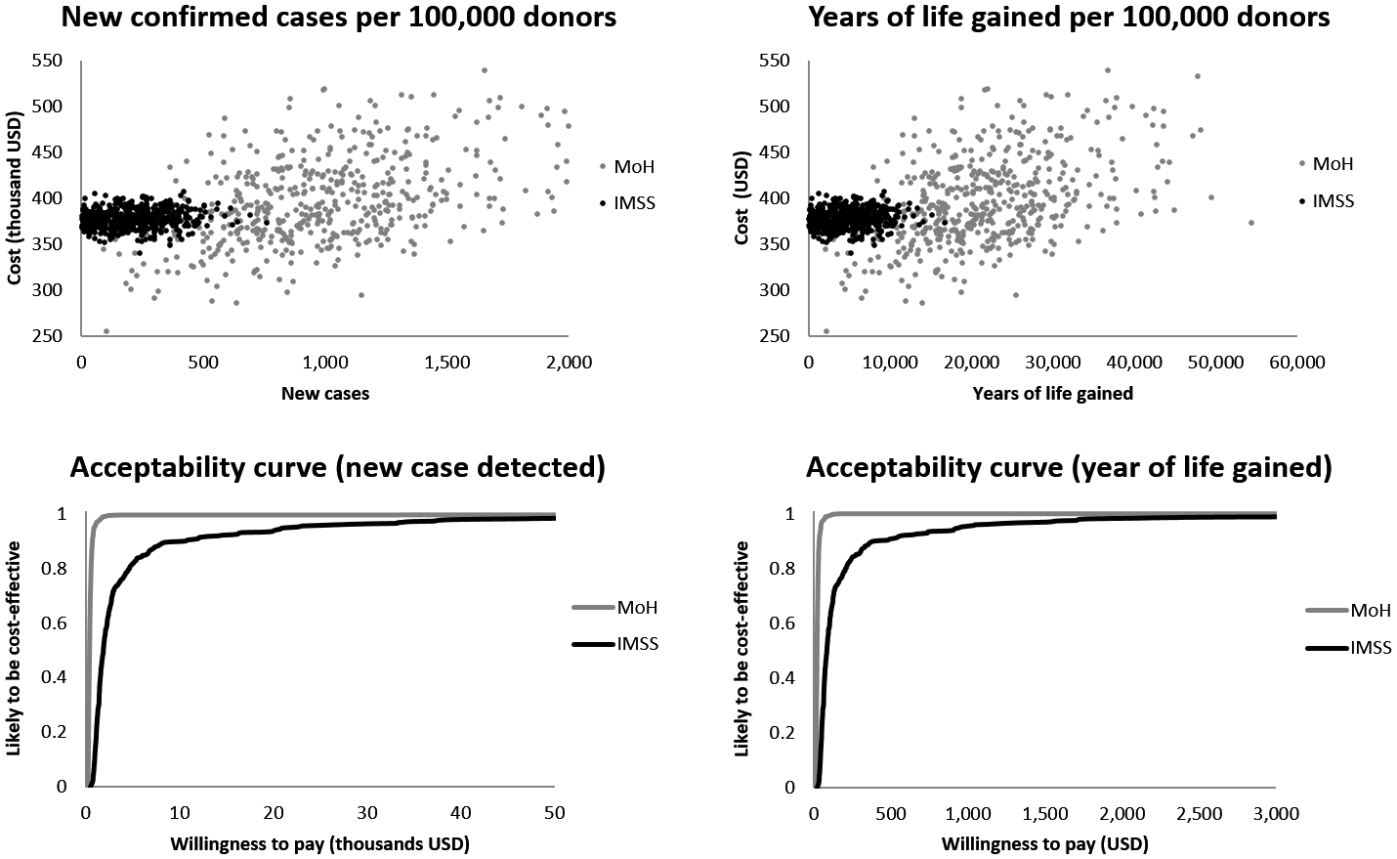
Acceptability curve for the willingness to pay per a year of life gained and per detected case for each alternative.

The incremental cost effectiveness ratio (ICER) for case detection by MoH is US$ 54,438 and US$ 383 for each life-year gained. The ICER for an additional case detected by IMSS is US$ 56,744 and US$ 463 for each additional life-year gained. The cost-effectiveness acceptability curves (CEAC) for the simulations suggest that willingness to invest is attractive above US$ 500 per year of life gained and US$ 8,000 per new case detected, based on 80% of cases falling below these thresholds (Fig 3). The Mexican government is willing to pay if the effectiveness unit is equivalent to the per capita value of the National Gross Domestic Product (GDP). The current Mexican GDP is approximately US$ 9,300, although a lower willingness to pay per unit of effectiveness is desirable for low and middle-income countries [46].

## Discussion

Serological screening of blood donations or donors for *T*. *cruzi* was mandated historically after 1990 in certain Latin America countries. Coverage of transfusion blood screening expanded to all Southern Cone Initiative countries after 1991, to some Central American countries after 1997, to most Andean Initiative countries after 1999, and to the Amazonian basin countries after 2004 [47–48]. Blood donation screening for *T*. *cruzi* in the United States became mandatory in 2011, before that in Mexico. Despite the fact that Mexico signed international agreements along with other countries and the World Health Organization to strengthen national blood banks and health policies to ensure safe blood supply, *T*. *cruzi* infected blood units were transfused in Mexico prior to 2007 with minimal blood screening (< 30%). Despite the fact that legislation for donation screening in Mexico was only approved finally in 2012, there is no information, monitoring or independent validation of screening compliance, or regarding infected-population follow-up. Incomplete compliance of Mexico’s national transfusion blood screening legislation affects costs and health outcomes, and hence should be analyzed using modified social and economic perspectives.

In 1991, a World Health Organization (WHO) expert committee recommended the use of either a single indirect hemagglutination test (IHA cutoff at 1:8) or a single latex agglutination test for donor or donation screening [49], while the Pan American Health Organization (PAHO) advocated in 1994 for the parallel use of at least two different serological tests for all donations [50]. However, in 2002, another WHO expert committee recommended a single enzyme-linked immunosorbent assay (ELISA) to screen blood donors or donations [51], while PAHO recommendations and other guidelines from Brazil [52], Chile [53], and Spain [54] suggested once again the use of two simultaneous different serological techniques run in parallel for *T*. *cruzi* screening (one of which should be an ELISA). The basis for this latter recommendation was that although ELISAs may occasionally give false positive results, they are the most sensitive, and confirmation could be run using a second confirmatory test [55]. Alternatives to existing serology have been developed and immunochromatographic test strips (ICS), also known as rapid tests, have recently been compared for primary healthcare level and blood bank use, given their lower cost and simplicity of use [56]. In most cases, rapid tests cost less than US$2 to the end user and a product cost of approximately US$0.25. However there have been few studies across indigenous and mestizo populations of Latin America to measure sensitivity, specificity, and agreement with existing serological assays, and none with joint analysis of cost and effectiveness [57]. Quantitative parasitological diagnosis of infection in patients is currently advancing rapidly with real time PCR [58–60], although validation needs to include all ethnic populations, infection and disease phases, and economic scenarios, according to targeted use (blood donation, early population-based diagnosis, chronic patients, congenital transmission, treatment efficacy). Current Brazilian guidelines recommend molecular screening only when serological tests are inconclusive [61].

Although control of *T*. *cruzi* transfusion transmission is an integral component of all CD prevention and control programs, few studies analyze costs or effectiveness of blood donor or donation screening, and none have analyzed both under different compliance scenarios. A Markov model has been used to estimate annual cost per person (US$ 4,660) and that for lifetime care (US$ 27,684) across countries with vector and non-vector transmission [62]. Bolivia established mandatory blood screening for HIV, hepatitis B, hepatitis C, and *T*.*cruzi*, and between 1996 and 2002, 11,489 *T*. *cruzi* -infected blood units were detected and discarded, and 2,879 potential infections prevented [63]. The cost of discarding one infected unit was US$ 96 and for preventing one potential infection was US$ 385. Blood donation screening to detect a positive CD case in Mexico is more expensive than in Bolivia, principally due to lower *T*. *cruzi* seroprevalence. The cost for preventing one additional potential infection in Mexico was estimated to be US$ 55,000 for the MoH, calculated along with social costs, the most important case cost component. It is important to note that blood product recipients are generally high risk, and may be even immunosuppressed, thereby having potentially early CD symptoms. In principle, these patients could be monitored, diagnosed and treated, which would lower cost estimates. However, in practice, *T*. *cruzi* infection induced by blood transfusion is not suspected or monitored due to lack of training or education regarding this neglected disease [11].

Mexican populations not included in this study were federal and state civil servants, public sector institutions (PEMEX), the armed services, and private health service providers. All but the latter two would have compliance equivalent to that of MoH, since MoH institutions are their primary provider of transfusion blood screening and confirmatory testing. The Mexican armed services and private health providers are reportedly screening blood donations at a rate similar to or greater than IMSS. Considering the number of blood units donated in 2012, and assuming equivalent compliance and a single blood unit per donor [64], present data indicate that during 2013 and 2014 incomplete compliance of national legislation by the MoH failed to confirm 15,162 *T*. *cruzi* infections, did not prevent 2,347 avoidable infections, and lost 333,483 life-years. The IMSS failed to confirm 2,184 *T*. *cruzi* infections, prevent 392 avoidable infections, and lost 47,986 life-years over the same two year period. Incomplete compliance and lack of oversight by the National Health Council for national blood transfusion legislation passively allows an avoidable economic burden for the population, principally due to work days lost. The current cost in Mexico due to healthcare per CD patient is around US$ 2,540, and the cost to the patient, due to work days lost, is approximately US$ 7,620 [11].

One of Mexico´s two principal health care institutions falls significantly short of blood donation screening compliance for *T*. *cruzi*, thereby affecting healthcare costs, case detection, and preventable life years. This study demonstrates that there is very little uncertainty that the decision to enforce complete compliance of blood donation screening is correct from a cost-effectiveness point of view. However, complete compliance will require unprecedented transparency of blood services´ information and rigorous monitoring programs for all healthcare institutions, particularly for reference networks and from government institutions. Until Mexico´s health, economy, and governance sectors recognize their responsibility for the continued burden of partial compliance of legislation, the Mexican population will continue to bear the weight of CD, and transmission risk will rise into the future.

## Supporting Information

S1 Table Blood bank survey conducted in 2007 of all registered centers within the National Center for Blood Transfusion (Centro Nacional de Transfusion Sanguinea, Mexico, CNTS). (XLSX)
